# Improvement in JOA Score of Treatment for Complex Atlas-Axis fractures

**DOI:** 10.12669/pjms.293.2995

**Published:** 2013

**Authors:** Yu Fu, Zhen-Ming Hu, Hong-jun Huo, Xuejun Yang, Ting Hao, Wan-lin Liu

**Affiliations:** 1 Yu Fu, Department of Orthopedics, Department of Orthopedics, The First Affiliated Hospital of Chongqing Medical University, Chongqing, China.; 2 Zhen-Ming Hu, Department of Orthopedics, Department of Orthopedics, The First Affiliated Hospital of Chongqing Medical University, Chongqing, China.; 3 Hong-jun Huo, Department of Spinal Surgery, The Second Affiliated Hospital of Inner Mongolia Medical University, Hohhot, China.; 4 Xue-jun Yang, Department of Spinal Surgery, The Second Affiliated Hospital of Inner Mongolia Medical University, Hohhot, China.; 5 Ting Hao, Department of Pediatric Orthopedics, The Second Affiliated Hospital of Inner Mongolia Medical University, Hohhot, China.; 6 Wan-lin Liu, Department of Orthopedics, The Second Affiliated Hospital of Inner Mongolia Medical University, Hohhot, China.

**Keywords:** Atlas, Axis, Atlanto-axial joint, posterior cervical pedicle screw fixation

## Abstract

***Objective:*** The aim of this study was to explore the role of treatment for complex Atlas-Axis fractures, and compare the JOA score of surgical and conservation methods.

***Methodology:*** From June 2008 to May 2012, 33 patients suffering from Atlas-Axis fracture were included in our study. Fifteen patients received posterior cervical pedicle screw fixation, and 18 patients received the conservation treatment. All the patients were followed up for 12 months after discharge.

***Results:*** The mean operative time was about 128 minutes (ranged: 92 to 165 minutes), the mean hospital stay time was 15.5 days (ranged: 8-21 days), and the mean follow-up of all the patients was 27months (ranged: 7 to 43 months). All patients gained a solid fusion, and no one showed any disability at the end of the follow-up. The JOA scores before treatment were 6.4±0.3 and 7.1±0.4 before and after treatment, and they significantly increased to 13.8±0.8 and 13.7±0.9 when following up for 12 months (*P*<0.05).

***Conclusions:*** Posterior cervical pedicle screw fixation is a feasible, effective and safe method for complex atlantoaxial fractures. This technique could achieve high JOA score, decreased blood loss and post-operative complications.

## INTRODUCTION

Complex Atlas-axis fractures often represent about 3% of all acute cervical spine injuries, 12% of upper cervical fractures,^1^^-^^3^ and it frequently occurs in elderly populaiton.^4^ Most of Atlas-axis fracture may be resulted from trauma in traffic accidents or a fall in elderly populations.^2^ Compared with the isolated atlas or axis fractures, complex Atlas-axis fractures have higher incidence of neurological impairment and mortality,^5^^-^^7^ and require early surgical stabilization. There is limited studies regarding on the treatment of Atlas-Axis fractures and there is no consensus between experts concerning treatment methods.^1^^,^^8^^,^^9^ The treatment principle is based on posterior internal fixation interlaminar clamps, and transarticular screw fixation. Fixation is required to reduce deformity, provide stability, and prevent impending neurological compromise till bony fusion occurs. Previous studies showed that the posterior cervical pedicle screw in the lateral mass of C1 and the pedicle of C2 was an option for atlantoaxial fixation.^10^^-^^12^ Nitising reported that posterior cervical pedicle screw is effective for obtaining fusion of the atlantoaxial complex, and the fusion rate are estimated to approach 100%.^12^ The posterior cervical pedicle screw can be incorporated as part of a modular system for fusions to the occiput and subaxial cervical spine, and thus the method could gain satisfactory results. 

There are few studies which have reported the role of posterior cervical pedicle screw in Chinese population. Therefore, we aimed to compare the effect of posterior cervical pedicle screw fixation with the conservative treatment in treatment of the Atlas-Axis fracture, and we evaluated the JOA scores of the treatment for the Atlas-Axis fracture.

## METHODOLOGY

***Patients: ***From June 2008 to May 2012, 33 patients suffering from Atlas-Axis fracture were included (18 males and 15 females; ranged 20-72 years old, mean age of 38.6±4.7. All the patients were confirmed by computed tomographic scans. The causes of instability were: injured by fall from height in eight patients, traffic accidents in 20 patients and failed previous surgery in five patients. Preoperative imaging included plain cervical radiographs with flexion and extension views, MRI and CT scans. All the patients did not have a neurological deficit. Written informed consent was obtained from all study subjects.

The preoperative and postoperative JOA scores (Japanese Orthopedic Association) and complications during the follow-up period were recorded. All the 33 patients showed posterior cervical pain in variable degrees of occipito cervical region and activity limitation. Five patients had difficulty in sensation, movement and reflex in the limbs. Our study was approved by the Ethics Committee of Inner Mongolia Medical University.

***Techniques:*** Posterior cervical pedicle screw fixation was used for cases with unstable C1-II odontoid fracture. A total of 15 patients received posterior cervical pedicle screw fixation. Patients with C1-CIII odontoid fracture and stable C1-hangman’s fracture were treated with conservative treatment, including skull traction, immobilization by hard cervical supporter, fixation by Halo frame. Finally 18 patients received the conservation treatment.

**Fig.1:**
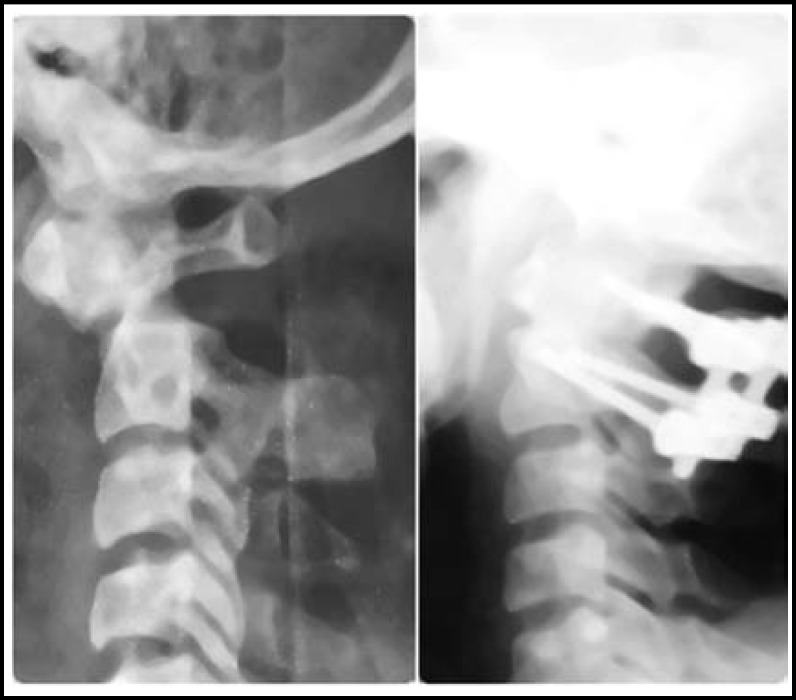
Patients before and after treatment by posterior cervical pedicle screw fixation

The patient was placed in prone position under general anesthesia. A midline incision was made to expose the posterior elements of C1 to C3, and then the atlanto-axial joint was exposed. The pedicle screw on atlas was placed at the intersection point, and it was about 20 mm beside the midpoint of atlantal posterior tubercle and 1-2mm superior to the inferior edge of posterior arch. By comparing with sagittal plane, the angle of the screw inclined to 0-5°, and the tip of the screw was placed at anterior arch, and it placed 5° to the head. After tapping, a 3.5-4.0mm multiaxial screw was inserted with the position of C-arm fluoroscopy. The entry point for the C2 pedicle screw was marked with a high-speed drill in the cranial and medial quadrant of the isthmus surface of C2. The pilot hole was drilled with a 2 mm bit, just perforating the opposite cortex, directing it 20–30ºC in a lateral-to-medial and cephalad trajectory. The integrity of the walls of the hole was verified with a ball probe. A 3.5 mm polyaxial screw was inserted. If necessary, the C1 ring was reduced by repositioning the patient’s head and/or directly manipulating C1 and C2 using the screws, followed by fixation to the rods to maintain alignment. If definitive fusion was required, autologous iliac crest cancellous bone was placed over the decorticated surfaces and wired into position (Fig.1).

***Follow-up: ***The drainage tube was removed 24-48 hours after fixation with cervical supporter. Patients were followed up with radiographs after 3 months, 6 months and 12 months. Dexamethason 10mg/time once a day and mannitol 250ml/time once a day were given by intravenous drip for cases with neural symptoms for three to seven days. Routine oral/parentral anti-inflammatory therapy was used for patients about three to seven days. The cervical supporter fixation was kept for patients about three months. An early postoperative CT scan was taken of all patients. During the follow-up at 3 months, we performed a CT scan for patients to understand the position of screw and bone fusion in atlantoaxial posterior surface after hospital discharge.

***Statistical analysis: ***Continuous variables were expressed as mean ± standard deviation (SD), while categorical variables were shown as frequencies and percentages. Statistical analysis of the data was performed by using χ^2^ test or student t test. P value less than 0.05 was considered significant.

## RESULTS

The mean operative time was about 128 minutes (ranged: 92 to 165 minutes), the mean hospital stay was 15.5 days (ranged: 8-21 days), and the mean follow-up of all the patients was 27months (ranged: 7 to 43 months). 15 patients who received posterior cervical pedicle screw fixation were regarded as surgery group, and 18 patients received conservation treatment as conservation group. The mean age of the surgery group and conservation group were 35.7±8.2 and 43.5±9.6 years, respectively, and there was significant difference between the two groups by student t test (t=2.4, *P*<0.05). No patient received blood transfusion during the surgery. In surgery group, seven patients were CI-CIII odontoid fractures, six patients were fracture-stable axis fractures and two patients were CI fracture-Hangman’s fractures. In the conservation group, 11 patients were CI-CIII odontoid fractures and four patients were C1 fracture-Hangman’s fractures. There was significant difference in the types of fractures between the two groups (χ^2^=7.6,* P*<0.05). During the follow-up period, no patient died or showed complications of the bone graft or screw rod. All patients gained a solid fusion, and no one showed any disability at the end of the follow-up.

Table-I shows the JOA scores of patients in the two groups during the follow-up period. The JOA scores in the conservation and surgical groups were 6.4±0.3 and 7.1±0.4 before treatment, and they increased to 13.8±0.8 and 13.7±0.9 when following up for 12 months, respectively (*P*<0.05). The JOA scores after treatment at the time of 3, 6 and 12 months in the two groups were significant higher than those before treatment (*P*<0.05).

**Table-I:** The JOA score in the two groups during the follow-up period

*Characteristics*	*JOA score*				*P value*
	*Before treatment*	*3 months *	*6 months*	*12 months*	
Conservation group	6.4±0.3	12.6±0.8	13.5±0.7	13.8±0.8	<0.05
Surgery group	7.1±0.4	13.1±0.6	13.3±0.5	13.7±0.9	<0.05

## DISCUSSION

The complex atlantoaxial fracture usually occurred in vehicle accident and fall in elderly patients.^4^^,^^13^^,^^14^ In our study, we collected eight patients injured by fall, 20 patients by traffic accidents and five patients failed in previous surgery. The most common atlas fracture is usually associated with odontoid fracture, ^4^^,^^15^ and we found 18 patients showed odontoid fracture in our study. The mechanism may be a sudden axial load inducing altas fracture.^4^

Another previous evidence showed the conservation treatment, such as skull traction, immobilization by hard cervical supporter, fixation by Halo frame, could gain good efficacy and few complications.^16^^,^^17^ However, we found that surgical treatment could achieve a similar efficacy and JOA score when compared with the conservation group. Because the cases in the surgical group had a serious fracture than those in conservation group, the surgical treatment had a better efficacy than the conservation group. Moreover, we did not find complications for patients received surgical treatment. 

Previous study showed that the operation could gain better improvement for patients with bone nonunion, unsuccessful reduction and maintenance of reduction, failure of the non-surgical management or inexact fracture of transverse ligament.^16^^,^^17^ Hein reported that altlantoaxial screw fixation used for unstable atlas burst fracture achieved a good bony fusion.^16^ Yamamoto reported two cases with atlas burst fracture who received surgical treatment gained a satisfactory results.^17^ Our results are in line with Hein and Yamamoto’s, and suggest the surgical results could gain a good results for the atlas fracture. 

Previous study reported that the pedicle screw fixation was an alternative surgical treatment for patients suffering from complex atlantoaxial fracture.^18^ Fielding reported C1-C2 pedicle screw fixation can achieve stability in all directions, and it also achieve a reliable axial stability.^18^ Eap reported that the three-dimensional stability of C1-C2 pedicle screws are comparable to the Magero screw, and the stability could be improved by combined with anterior cervical odontoid screw fixation.^19^ Andersson reported pedicle screw fixation is biomechanically rigid and prevent the rotation of the atlantoaxial complex as well as provides immediate multidirectional stability.^20^

The choice of treatment for complex atlantoaxial fractures is usually determined by stability of spine and injury of bone ligament. Patients suffering from complex atlantoaxial fracture complicated with C1/2 or C2/3 instability should receive early surgery rather than other types of axis fracture. Moreover, surgical treatment must be performed for patients with C1 fracture complicated with type II odontoid fracture and atlas transverse ligament rupture.^21^ If C1 posterior arch is absent or unstable, posterior C1-C2 pedicle screws, transarticular screw or occipital-cervical fusion could be used during surgery.^21^

This study showed posterior cervical pedicle screw fixation is a feasible, effective and safe method for complex atlantoaxial fractures, this technique could avoid less trauma, decreased blood loss and post-operative complications. Further large sample studies on the effectiveness of internal fixation are greatly needed. 

## Authors Contribution

Yu Fu, Zhen-Ming Hu, and Ting Hao contributed to the study design, conduction, and paper writing. Zhen-Ming Hu and Wan-lin Liu contributed to the study design. Yan-feiJia, Zeng-taoHao and Yan-xiang Tong contributed in conducting the study.
